# Comparative treatment planning of very high-energy electrons and photon volumetric modulated arc therapy: Optimising energy and beam parameters

**DOI:** 10.1016/j.phro.2025.100732

**Published:** 2025-02-16

**Authors:** Fabio S. D’Andrea, Robert Chuter, Adam H. Aitkenhead, Ranald I. MacKay, Roger M. Jones

**Affiliations:** a The University of Manchester, Physics and Astronomy, Manchester, United Kingdom; b The Cockcroft Institute, Accelerator Science and Technology, Daresbury, United Kingdom; c The Christie NHS Foundation Trust, Christie Medical Physics and Engineering, Manchester, United Kingdom; d The University of Manchester, Division of Cancer Sciences, Manchester, United Kingdom

**Keywords:** VHEE, TPS, Treatment planning, Monte Carlo, Very high-energy electron, Radiotherapy, matRad, VMAT, FLASH

## Abstract

**Background:**

Very High-Energy Electron (VHEE) beams offer potential advantages over current clinical radiotherapy modalities due to their precise dose targeting and minimal peripheral dose spread, which is ideal for treating deep-seated tumours. To aid the development of clinical VHEE machines, this study adressed the need to identify optimum VHEE beam characteristics for tumours across various anatomical sites.

**Materials and methods:**

VHEE treatment planning employed matRad, an open-source treatment planning system, by adapting its proton pencil beam scanning implementation. VHEE beam characteristics were generated using TOPAS Monte Carlo simulations. A total of 820 plans were retrospectively created and analysed across 10 pelvic and 12 thoracic cases and compared against clinical photon VMAT plans to identify the most optimal VHEE beam configuration and energy requirement.

**Results:**

VHEE plans outperformed photon VMAT in sparing organs-at-risk (OARs) while maintaining or improving target coverage. While 150 MeV served as the threshold for effectively treating deep-seated sites, 200 MeV was identified as a more optimal energy in the pelvis for achieving the best balance of penetration and sparing abutting OARs. Lower energies (70–110 MeV) also benefitted mid-to-superficial disease in the lung cohort. Typically, VHEE plans required 3–5 fields, and resulted in notable dose reductions to OARs across treatment sites, including: 22.5% reduction in rectal D_mean_; 13.8% decrease in bladder D_mean_; 8.2% reduction in heart D_mean_; and a 24.4% decrease in lung V_20Gy_.

**Conclusion:**

The study reinforces VHEE’s potential in clinical settings, emphasising the need for varied energy ranges to enhance treatment flexibility and effectiveness.

## Highlights

• matRad, an open-source planning system adapted for Very High Energy Electrons (VHEE).
• VHEE plans show dosimetric advantages over photon arc therapy in two clinical sites.
• Data from 820 plans and 22 patients reveals trends in energy and beam delivery.
• Threshold at 150 MeV, 200 MeV optimal − 110 MeV for superficial disease.
• Three to five fields typically required for VHEE plans to achieve these goals.

## 1 Introduction

Very High Energy Electrons (VHEE), characterised by energies above 50 MeV, have emerged as a promising radiotherapy modality, with the potential to precisely target deep-seated tumours while minimising peripheral dose spread [1], [2]. VHEE beams offer efficient dose delivery and, with magnetic steering, may enable ultra-high dose rates, reducing the need for extensive collimation or mechanical adjustments [3], [4].

Despite these advantages, crucial questions remain. Studies suggest that scanned VHEE beams can achieve dose distributions comparable or superior to photon and proton therapies [5], [6], [7], [8], even with simpler conformal planning techniques [9]. In the absence of a clinical VHEE machine, efforts to optimise treatment planning and fully evaluate its clinical potential remain theoretical, necessitating comprehensive studies to establish key delivery parameters. One critical consideration is beam energy selection. Higher-energy VHEE beams are often favoured for their reduced lateral dose spread and deeper penetration [1], [2], [10], but undesired distal dose deposition beyond the target is a drawback. In contrast, lower-energy VHEE beams (70–110 MeV) exhibit a steeper longitudinal dose fall-off, potentially improving dose conformity in specific clinical scenarios. These trade-offs highlight the need for tailored planning strategies to optimise VHEE for diverse clinical scenarios.

To fully exploit VHEE’s potential and deliver tailored treatments at ultra-high dose rates necessary to induce the FLASH effect [11], [12], [13], machine designs must address both the need for variable energy configurations and efficient treatment delivery through fixed beam lines [14], [15]. Determining the optimal number of beams remains a key challenge [16], [17].

This study aims to provide a detailed and generalisable assessment of the dosimetric properties of VHEE beams across two key anatomical sites, utilising a large sample of patients and multiple plan iterations. The analysis focuses on determining the optimal beam number, energy requirements (including the comparative dosimetric roles of different VHEE energies), and spot spacing by comparing retrospective VHEE plans with clinical photon VMAT plans. By examining treatments for two patient cohorts, this study seeks to advance the clinical viability of VHEE by maximising therapeutic outcomes and informing the development of VHEE delivery systems.

## 2 Materials and methods

This study employed matRad (v2.10.1) [18], a multimodal open-source research treatment planning system (TPS) developed by the German Cancer Research Centre (DKFZ) for photon and hadron therapy. matRad is written in MATLAB (R2023b, The MathWorks, Natick, Massachusetts).

### 2.1 VHEE beam modelling

matRad requires beam data as a function of depth in water to calculate patient dose. Beam data were generated using Monte Carlo (MC) simulations with TOPAS (v3.8.1) [19], a Geant4 [20] wrapper, in a water phantom. Simulations featured monoenergetic VHEE beams with symmetrical Gaussian distributions at 70, 110, 150, 200, and 240 MeV. These energies represent a feasible range for compact RF solutions (e.g. X‐band linacs, gradients≈100 MeV/m [21]), maintaining a compact footprint for clinical integration.

Beam sizes, with σ values of 2, 4, 11, 21, and 42 mm, were scored on a 1 mm^3^ grid. The smaller (2–4 mm) spots were similar to those used in proton spot scanning, while the larger spots resemble conformal photon beams. This σ range aligns with achievable beam optics and underpins treatment planning [22]. Histories were adjusted by beam size to ensure consistent fluence, with σ = 4 mm utilising 10^7^ electrons. Electrons were transported in vacuum to the surface of a homogeneous water phantom, where integrated depth dose profiles formed the basis of matRad’s beam model.

### 2.2 matRad workflow and dose calculation

Details of matRad and its validation are outlined elsewhere [18]. Although matRad does not natively support VHEE, its proton scanning infrastructure was modified in-house for VHEE planning. The VHEE implementation employed an active spot‐scanning technique similar to that used for protons [23]. In contrast to protons, which require multiple energy layers for a homogeneous dose via a Spread‐Out Bragg Peak (SOBP) [24], VHEE plans employed a single energy layer. Dose calculations used a conventional dose‐to‐water pencil beam algorithm derived from energy‐dependent custom base data from TOPAS [25], [26]. Water‐equivalent lengths [27] were determined for each pencil beam, and dose deposition per voxel was calculated as the product of the longitudinal depth‐dependent deposition and a lateral Gaussian component.

### 2.3 Patient selection

22 patients were selected across two cohorts—10 prostate and 12 lung—representing varied tissue densities, tumour sizes and depths. All had previously received photon VMAT (planned using Pinnacle v16.0 (Philips, Amsterdam, Netherlands)) and served as the reference. Local approval was granted, and written informed consent was obtained. Prostate plans received 60 Gy in 20 fractions, with the Planning Target Volume (PTV) mean dose at 100%. Lung plans received 55 Gy in 20 fractions, with the Internal Target Volume (ITV) mean dose at 100%. Representative PTV slices appear in Supplementary Figs. 1 and 2.

### 2.4 Dose grid and spot spacing

All plans used a 2×2×2 mm^3^ dose grid. Spots were placed on a square grid covering the PTV projection in the beam's-eye-view (BEV) with a 5 mm margin. This spacing balanced hardware demands and planning time while preserving target coverage. To validate spot spacings, three lung patients (with the largest, smallest and median PTVs) were sampled to inform the broader study.

### 2.5 VHEE treatment planning

An automated treatment planning procedure was employed using in-house scripts, minimising operator bias and enabling systematic evaluation of varying VHEE beam energy, number of beams, and spot spacing. The automation pipeline comprised three stages: beam configuration, dose calculation, and plan optimisation. First, the script imported patient imaging data and assigned beam configurations based on pre-specified angular arrangements (Section 2.5.1) and corresponding energy (Section 2.5.2). Next, an influence matrix was calculated with matRad to model dose distributions, then utilised in its optimisation to generate plans that met consistent clinical goals. Dose-volume constraints were determined via a protocol-driven approach informed by data and guidelines from relevant clinical trials and consensus protocols, ensuring adherence to evidence-based practices. Constraints were applied uniformly to both VHEE and reference photon plans for direct comparison. A total of 820 VHEE plans were generated, representing a comprehensive analysis of the parameter space.

#### 2.5.1 Beam arrangement

Lung cases were planned with single-beam, parallel-opposed, three, five, seven, nine, and 12 beams. Plans with five beams or more used equidistant angles spanning 200°, mirroring IMRT strategies, with the 12-beam plan approximating a pseudo-VMAT. One- to three-beam plans explored feasibility in low-beam scenarios, important for FLASH.

Prostate configurations were similar but spanned 360°, including up to 15 beams. Examples of each arrangement for both cohorts are shown in Supplementary Figs. 3 and 4.

#### 2.5.2 Energy requirements

To assess energy requirements for generating clinically acceptable plans, each VHEE beam configuration was planned five times using energies of 70, 110, 150, 200, and 240 MeV. These energies were selected due to the significant variations in dose deposition associated with each energy.

## 3 Results

### 3.1 VHEE properties

**Fig. 1:**
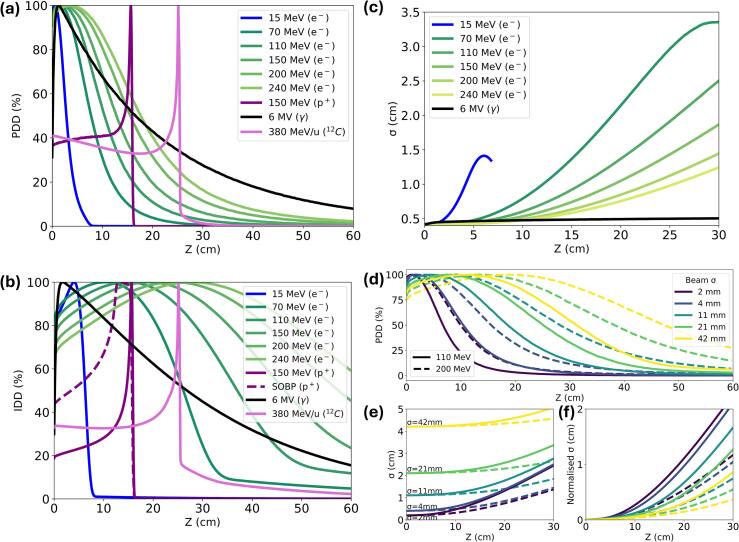
**Monte Carlo simulations comparing VHEE to clinical modalities.** This figure presents MC simulations comparing Gaussian VHEE beams, with a beam σ of 4 mm unless specified otherwise, to conventional clinical radiotherapy modalities. The depth (z) in water is plotted on the x-axis across all subplots. Evaluation metrics include (a) central-axis Percentage Depth Dose (PDD) curves, (b) Integrated Depth Dose (IDD) curves, (c) variation of beam size (σ) as a function of depth, and (d) the influence of initial spot size on beam broadening for 110 and 200 MeV VHEE beams, (e) the influence of initial spot size on the variation of σ as a function of depth for different initial beam σ, and (f) normalised σ as a function of depth for different initial spot sizes.

The Percentage Depth Dose (PDD) curves (Fig. 1a) showed that clinical electron beams rapidly achieved peak dose at shallow depths, while VHEE beams penetrated deeper, benefiting treatment of deep‐seated tumours. VHEE beams preserved a greater proportion of the maximum dose at depth, as shown by the Integrated Depth Dose (IDD) curves (Fig. 1b) featuring an extended plateau. Furthermore, VHEE beams showed reduced lateral spread (σ) when propagating through water (Fig. 1c).

The effect of initial spot size on dose distribution and lateral spread was analysed using large‐aperture beams (e.g., σ = 42 mm, corresponding to 10 cm FWHM) to compare field sizes, rather than any specific clinical configurations. Larger initial spot sizes produced a shallower dose fall‐off than smaller spots at both 110 and 200 MeV (Fig. 1d). Furthermore, larger spot sizes incurred a reduced increase in lateral spread at depth (Fig. 1e). The σ, normalised to initial spot size (Fig. 1f), supported this trend for both 110 and 200 MeV, with higher‐energy beams sustaining a tighter lateral spread for all spot sizes.

### 3.2 Spot spacing and matRad performance

**Fig. 2:**
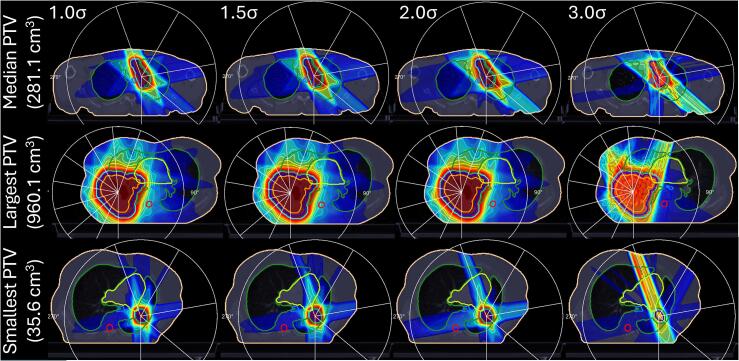
**Impact of spot spacing optimisation using VHEE for lung cancer**. Three patients are shown, representing the largest, smallest, and median PTV volumes. Spot spacings of 1.0, 1.5, 2.0, and 3.0σ are assessed, with examples from five-beam and nine-beam plans utilising 200 MeV shown. All plans were generated with a spot size of σ = 4 mm.

A targeted lung cohort sample validated the spot spacing for subsequent lung and prostate cases. Fig. 2 illustrates dose distributions in three lung cases with varied PTVs using three-, five- and nine-beam configurations. The aim was to balance hardware utilisation and planning time without compromising target coverage. Additionally, matRad’s dose calculation was benchmarked against MC simulations (Supplementary Fig. 5).

Visually, expanding spot spacing from 1.0σ to 3.0σ reduced dose uniformity within each PTV. The change was minimal between 1.0σ and 1.5σ but pronounced at 3.0σ, where no viable plan was produced.

Quantitatively, PTV D_95%_ decreased with increasing spot spacing, culminating in a 5.1% reduction at 3.0σ relative to 1.0σ, while PTV D_1cm_^3^ increased. The Homogeneity Index (HI) deteriorated, confirming reduced uniformity (Supplementary Table 1). Adjusting spacing from 1.0σ to 1.5σ preserved dose homogeneity and coverage, with minimal differences in PTV D_95%_ and D_1cm_^3^ (0.2% and 0.4%, respectively). This strategy reduced computational demands by decreasing spots per 100 cm^3^ of PTV from 422 to 208 and halving the mean planning time.

### 3.3 Prostate

**Fig. 3:**
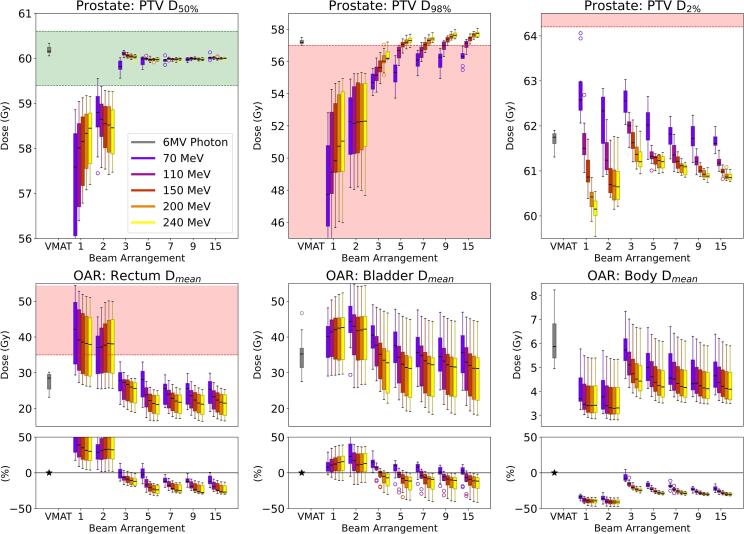
**Comparison of dose distributions in prostate cancer treatment.** The figure displays box-and-whisker plots comparing 420 dose distributions between 6 MV Photon VMAT and VHEE across various beam numbers (single, parallel-opposed, 3, 5, 7, 9, 15) and energies (70, 110, 150, 200, and 240 MeV) based on data from 10 patients. The VHEE plans were generated with a spot size of σ = 4 mm and a spot spacing of 1.5σ. It presents PTV statistics (D_50%_, D_98%_, D_2%_) and dosimetric outcomes (D_mean_) for critical OARs: rectum, bladder, and body. Outliers are represented as individual points. Additionally, difference plots are included to visualise dosimetric deviations for OARs between the two modalities, highlighting relative and absolute differences across configurations. The green shaded areas represent the target dose coverage goals for the PTV, while the red shaded areas indicate the dose constraints for the respective volumes. (For interpretation of the references to colour in this figure legend, the reader is referred to the web version of this article.)

For the PTV, VHEE plans with three or more beams achieved similar doses to the VMAT reference plans for PTV D_50%_, aligning with the 60 Gy prescription (Fig. 3). Higher energies and additional beams improved conformity. Single-beam and parallel-opposed plans exhibited marked variability and frequently did not meet the prescription, while plans with five or more beams consistently approximated the reference dose. For PTV D_98%_, higher energy VHEE beams (≥150 MeV) reliably delivered the minimum PTV dose, especially with five or more beams. Lower energy plans (70–110 MeV) and those with fewer beams failed to meet clinical goals. PTV D_2%_ varied with energy, as lower energies delivered higher doses than the VMAT reference. Increasing the beam number and energy reduced the maximum dose, either improving upon or equalling the VMAT plans and indicating a more uniform distribution.

For organs-at-risk (OARs), the rectal D_mean_ significantly decreased with additional beams and higher energies. Plans with three or more beams exhibited lower rectal doses; those with five or more beams achieved reductions of up to 22.5% relative to the VMAT reference. Higher energy beams (≥150 MeV) further reduced the rectal D_mean_ compared with VMAT. Similarly, the bladder D_mean_ decreased slightly relative to VMAT, with reductions more dependent on beam number than energy. Configurations with five or more beams reduced the bladder D_mean_ by up to 13.8%. Additional OARs, including the bowel, and additional statistics for the rectum and bladder showed similar trends (Supplementary Fig. 6). For total body dose, higher energies clearly reduced mean body doses, particularly with more than five beams, indicating that higher energy VHEE treatments possess the ballistic properties required for conformal dose distribution at depth.

### 3.4 Lung

**Fig. 4:**
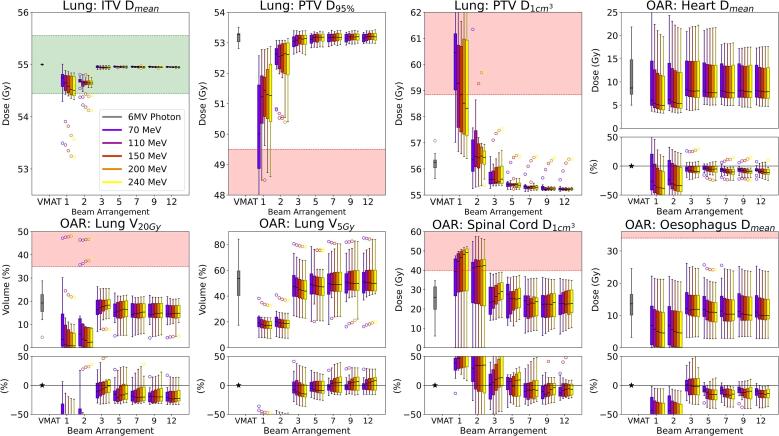
**Comparison of dose distributions in lung cancer treatment**. The figure displays box-and-whisker plots comparing 400 dose distributions between 6 MV Photon VMAT and VHEE across various beam numbers (single, parallel-opposed, 3, 5, 7, 9, 12) and energies (70, 110, 150, 200, and 240 MeV) based on data from 12 patients. The VHEE plans were generated with a spot size of σ = 4 mm and a spot spacing of 1.5σ. It presents target volume statistics (ITV D_mean_, PTV D_95%_, and PTV D_1cc_) and dosimetric outcomes for critical OARs: Lung V_20Gy_ & V_5Gy_, Heart D_mean_, Oesophagus D_mean_ and Spinal Cord D_1cm_^3^. Outliers are represented as individual points. Additionally, difference plots are included to visualise dosimetric deviations for OARs between the two modalities, highlighting relative and absolute differences across configurations. The green shaded areas represent the target dose coverage goals for the ITV, while the red shaded areas indicate the dose constraints for the respective volumes. (For interpretation of the references to colour in this figure legend, the reader is referred to the web version of this article.)

As shown in Fig. 4, the mean dose to the ITV (D_mean_) displayed minimal dose variation across various VHEE energies and beam configurations, except in single-beam and parallel-opposed setups. Plans utilising three or more beams consistently achieved the prescribed 55 Gy, closely matching the dose coverage provided by the 6 MV photon VMAT plan. In multi-beam configurations, the PTV D_95%_ maintained consistent dose coverage, improving with additional beams. However, dose uniformity revealed a significant trade-off. Multi-beam setups achieved optimal D_1cm_^3^, comparable to or surpassing those of VMAT, while single-beam and parallel-opposed configurations demonstrated diminished uniformity. This underscores the capability of multi-beam VHEE plans to improve dose uniformity in lung treatments across various energies.

Increasing the number of beams from three to twelve markedly enhanced healthy lung tissue preservation, with an average reduction in lung V_20Gy_ of 24.4% across all scenarios compared to the photon VMAT plans. This trend persisted across all energies and was accompanied by an average 8.2% reduction to heart D_mean_ in multi-beam configurations. Additionally, spinal cord and total body doses decreased with an increase in the number of beams, offering superior dose statistics relative to VMAT, particularly with five to twelve beams.

#### 3.4.1 Energy effects by anatomical location

**Fig. 5:**
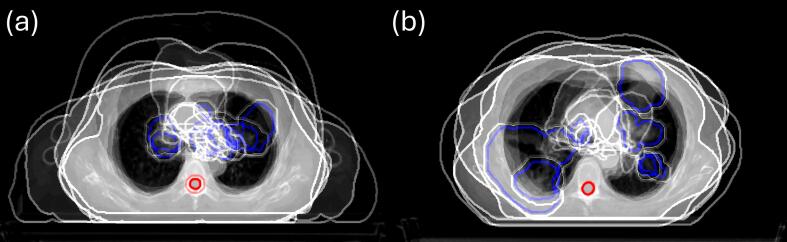
**Superimposed breakdown of the lung cohort depicting PTV (blue contours) locations matched to the spinal cord.** (a) displays PTVs overlapping with the heart in the midline (n = 7, median PTV: 206.7 cm^3^), while (b) shows PTVs near the chest wall, away from the midline, with reduced proximity to critical structures (n = 5, median PTV: 271.5 cm^3^). (For interpretation of the references to colour in this figure legend, the reader is referred to the web version of this article.)

While the prostate cohort exhibited significant sensitivity to energy, the same methodology applied to the lung cohort showed minimal dependence on VHEE energies. To investigate further, the lung cohort was separated based on PTV positioning into two groups: midline PTVs overlapping critical structures (e.g. heart, oesophagus), and PTVs near the chest wall (Fig. 5).

For midline PTVs, VHEE plans with energies from 110 to 200 MeV reduced normal tissue exposure relative to conventional photon VMAT treatments. At 110 MeV, lung doses decreased by 6.7% in V_5Gy_ and 18.2% in V_20Gy_ relative to photon benchmarks. As energy increased to 150 and 240 MeV, improvements in V_5Gy_ persisted, while V_20Gy_ reductions stabilised between 11.0% and 12.9%, suggesting a plateau at higher energies. Moreover, D_mean_ to the oesophagus and heart decreased significantly, with reductions up to 30% for the oesophagus and 5.6–9.3% for the heart at 200 MeV (see Supplementary Figs. 7 and 8 for cohort analyses).

Conversely, PTVs adjacent to the chest wall benefited from lower VHEE energies (70–150 MeV). At 70 MeV, lung V_20Gy_ decreased by 30% and spinal cord D_1cm_^3^ by 16%, owing to favourable longitudinal dose fall-off. Reductions in oesophagus and heart D_mean_ (10.8–14% between 110 and 150 MeV) further support the use of mid-range energies for lateral PTVs. However, as energy surpassed 150 MeV, improvements for the heart and oesophagus were marginal, with only a 2% further decrease in heart dose offset by a slight increase in spinal cord and lung V_20Gy_.

## 4 Discussion

VHEE was retrospectively compared with clinical photon VMAT plans for lung and prostate cancer to assess dosimetric properties, beam configurations and energy requirements, and to highlight potential advantages and limitations.

MC simulations demonstrated that VHEE yields deeper, more uniform dose distributions than conventional electron and photon beams, rendering it suitable for deep‐seated tumours. The PDD and IDD curves for Gaussian beams agree with large‐field studies [28], while extending exploration to smaller spot sizes for scanning. Spot sigma values of 2–4 mm reflect those produced by modern RF‐driven systems [5], [22]. Although smaller sigma values may enhance conformity, excessively small values compromise stability by necessitating additional spots, increasing complexity, intensifying scattering, and raising computational demands. Thus, σ = 4 mm was chosen as a practical balance.

Although the matRad analytical model delivers sufficient accuracy for VHEE dosimetry, caution is warranted in heterogeneous media, including air and bone. Its speed enabled a comprehensive parameter study across a patient cohort, impractical with MC methods alone. Continued improvements in GPU‐based MC methods [29] may allow future studies to achieve similar efficiency with enhanced accuracy in complex heterogeneous tissues. Pencil beam modelling excluded angular and energy spread in air and treatment head contamination, which may affect surface dose and beam characteristics at lower energies. At 70  MeV air scattering increases, affecting beam size and resulting dose distribution; however, these effects remain minor relative to scattering in the treatment medium [1]. Although such factors may influence dose distribution, they are unlikely to alter overarching conclusions. In treating lung and prostate diseases, VHEE achieved dose conformity competitive with, and sometimes superior to, state‐of‐the‐art VMAT implementations. Nonetheless, challenges and limitations remain.

A limitation of previous VHEE treatment planning studies is the focus on individual dosimetric differences [5], [6], [7], [8], typically based on a single case per anatomical site. While this approach permits exploration of dosimetric variations across treatment sites, it does not incorporate a large‐sample analysis. Such analysis is crucial for evaluating average differences in dose metrics across patient cohorts, thereby providing generalisable insights.

In the prostate cohort, results indicated a strong dependency on energy and beam number. A threshold of 150 MeV was required; below this, plans lacked adequate coverage. Optimal outcomes occurred at 200 MeV using five or more beams. Under these conditions, target coverage was met or exceeded, yielding superior dose uniformity relative to VMAT. Increasing beam number and energy significantly reduced mean rectal and bladder doses. This suggests that utilising multiple VHEE beams improves plan quality relative to current megavoltage photon VMAT techniques.

In the lung cancer cohort, variability in tumour size, location, and proximity to OARs poses challenges in establishing trends in the heterogeneous thoracic region. VHEE plans employing three or more beams provided adequate dose coverage and distribution with minimal energy dependency. Even the lowest tested energy of 70 MeV achieved sufficient penetration and conformality. Moreover, using fewer beams facilitates FLASH radiotherapy [30] by enabling rapid dose delivery, minimising interruptions, simplifying planning, and maintaining dose-rate thresholds. VHEE plans demonstrated superior sparing of normal tissues, especially in the lung, oesophagus, and heart, highlighting their capacity to protect critical structures. A distinct advantage of VHEE, not examined in this study, is its reduced sensitivity to inhomogeneities [31]. This trait may prove invaluable in lung cancer cases involving mobile structures, where preserving healthy lung tissue and cardiac function is paramount.

The analysis identified site-dependent energies, namely 110–200 MeV for midline-based PTVs and 70–150 MeV for PTVs adjacent to the chest wall. Notably, 150 MeV was optimal for both sub-groups, balancing dose reduction to normal tissue with effective target coverage. Additional energies—specifically 70 and 110 MeV for rapid longitudinal dose fall-off, and 200 MeV to minimise lateral spread in cases with overlapping or adjacent OARs—further enhance treatment flexibility.

Previous studies have highlighted the advantages of higher VHEE energies (100–250 MeV) in achieving superior conformity and dose distributions for deep-seated tumours, while reducing integral dose compared to VMAT and intensity-modulated proton therapy (IMPT) [5], [6], [10]. However, limited benefits were reported for lower energies due to reduced penetration and dose conformity [7].

Our findings align with the benefits of higher energies and demonstrate the utility of lower-energy VHEE beams (e.g., 110 MeV) in scenarios requiring precise dose fall-off near critical structures or superficial sites. This suggests that the potential of lower-energy beams in specific anatomical contexts may have been underexplored in previous research. Incorporating a broader spectrum of VHEE energies into machine design could expand treatment options, by enabling customised dose distributions that maximise tumour targeting while sparing nearby OARs. These results complement earlier studies by demonstrating the diverse applications of VHEE energies, including the distinct advantages provided by lower energies in specific cases.

A limitation of this study is that, due to the exploration of numerous plans, VHEE plans did not always achieve the most optimal solution for each scenario, in contrast to the individually optimised clinical photon plans used for benchmarking. Although a class solution approach was employed to harmonise energies and beam arrangements, it may introduce operator bias favouring VMAT over script‐based VHEE plans. Consequently, actual VHEE performance might exceed that demonstrated, and this bias should be considered when interpreting the findings. Future improvements may include mixed energy beams, optimised beam angles, non‐coplanar geometries, and focusing techniques to further enhance plan quality [22], [32]. This research lays the groundwork for future studies on patient heterogeneities, biological functions, and experimental validation of VHEE.

Translating VHEE therapy from theoretical models to clinical practice presents significant challenges alongside promising opportunities. The primary hurdle is the lack of commercially viable VHEE machines, which hampers clinical trials essential for regulatory approval and adoption. Nonetheless, simulation and theoretical evidence underscore VHEE’s potential advantages. Incorporating a range of energies—especially 110, 150, and 200  MeV—may enhance treatment flexibility. Several facilities are proposed [33] or under development, such as FLASHDEEP, intended to be the first to deliver FLASH radiotherapy using VHEE beams [34].

Our analysis of 820 retrospective VHEE plans across 22 patients indicates that effective dose distributions are achievable with as few as three to five beams. These findings further emphasise the need for a spectrum of energies: a minimum threshold of ≥150  MeV, an optimal 200  MeV for deep-seated disease in pelvic sites and for targets near critical organs, and a lower energy option such as 110  MeV for superficial disease, as demonstrated in lung cases. The versatility of VHEE technology supports its application across multiple anatomical regions, contingent on the development of appropriate delivery systems. These results establish a threshold that ensures technologies remain affordable without compromising treatment quality.

## CRediT authorship contribution statement

**Fabio S. D’Andrea:** Conceptualization, Methodology, Resources, Software, Validation, Formal analysis, Investigation, Data curation, Visualization, Writing – original draft, Writing – review & editing, Visualization. **Robert Chuter:** Conceptualization, Resources, Data curation, Writing – review & editing, Supervision. **Adam H. Aitkenhead:** Conceptualization, Writing – review & editing, Supervision. **Ranald I. MacKay:** Conceptualization, Writing – review & editing, Supervision. **Roger M. Jones:** Conceptualization, Writing – review & editing, Supervision, Funding acquisition, Project administration.

## Funding

This research was funded by the 10.13039/100010595Cockcroft Institute 10.13039/100014988of Science and Technology 10.13039/501100000271STFC grant: (ST/V001612/1). RC was supported by the 10.13039/501100017008Cancer Research UK Manchester Centre award [CTRQQR-2021\100010]. AA was supported by 10.13039/501100000289Cancer Research UK via the funding to 10.13039/501100017008Cancer Research UK Manchester Centre: (C147/A18083) and (C147/A25254).

## Declaration of competing interest

The authors declare that they have no known competing financial interests or personal relationships that could have appeared to influence the work reported in this paper.
