# Supplementary material for: Comparative treatment planning of very high-energy electrons and photon volumetric modulated arc therapy: Optimising energy and beam parameters
Source: Phys Imaging Radiat Oncol. 2025 Feb 16;33:100732. doi: 10.1016/j.phro.2025.100732 (PMC11926434; doi:10.1016/j.phro.2025.100732)
Supplement: Supplementary Data 1 [file mmc1.pdf]

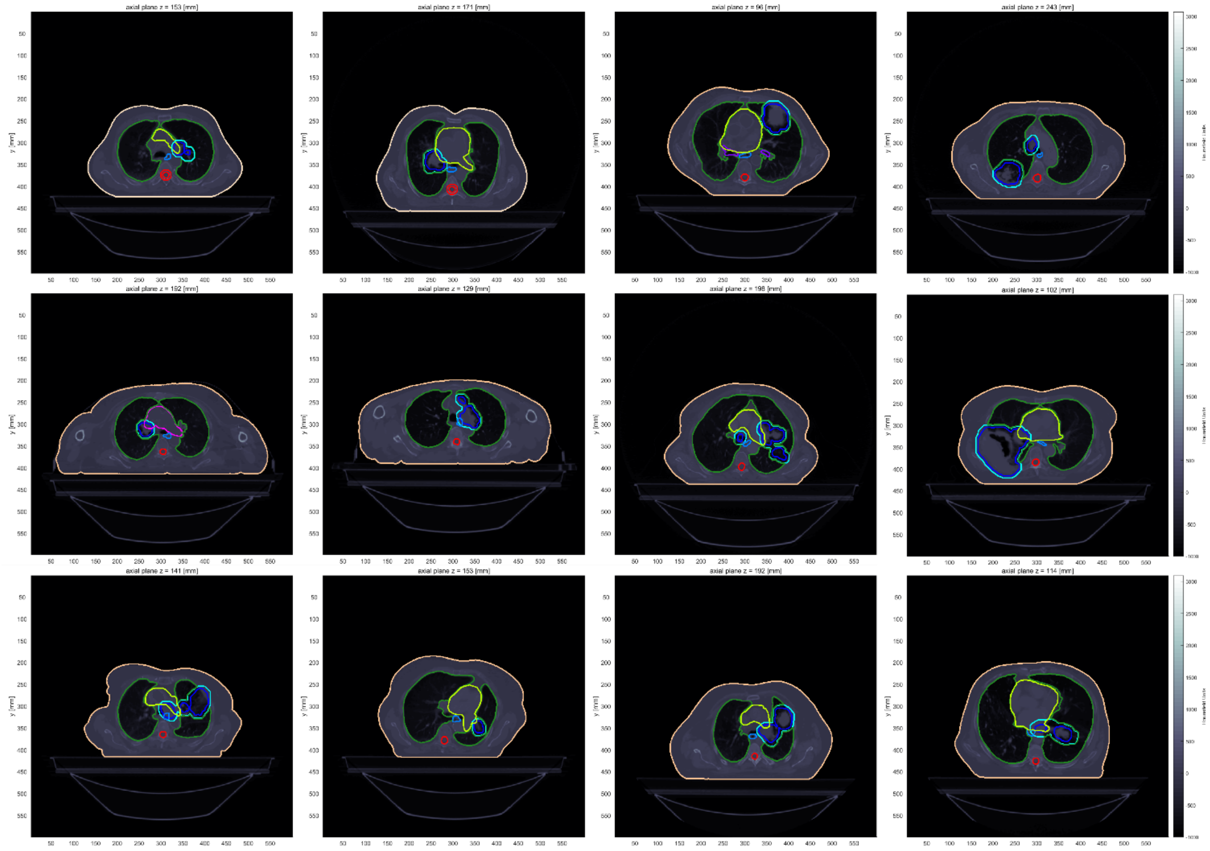

Supplementary Figure 1: Representative slices through the Planning Target Volume (PTV) for 12 Lung Cases.

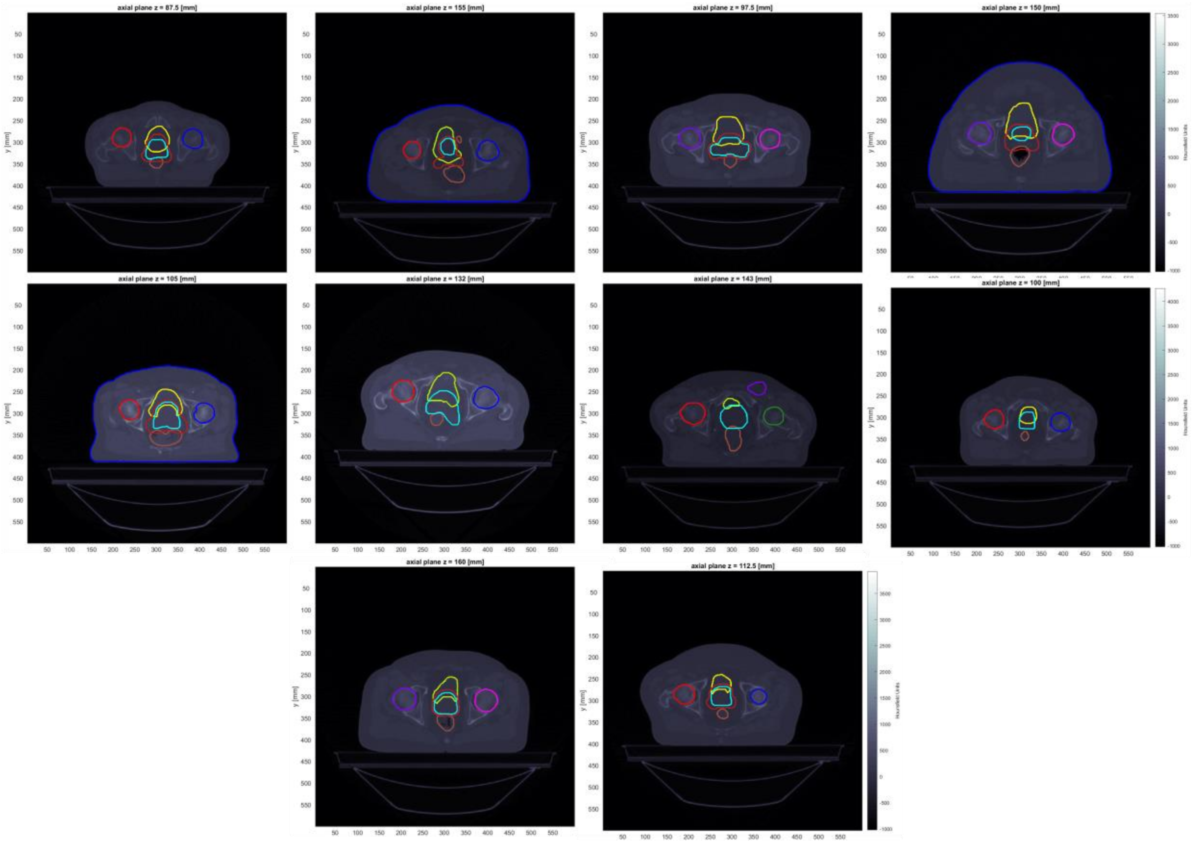

Supplementary Figure 2: Representative slices through the Planning Target Volume (PTV) for 10 Prostate Cases

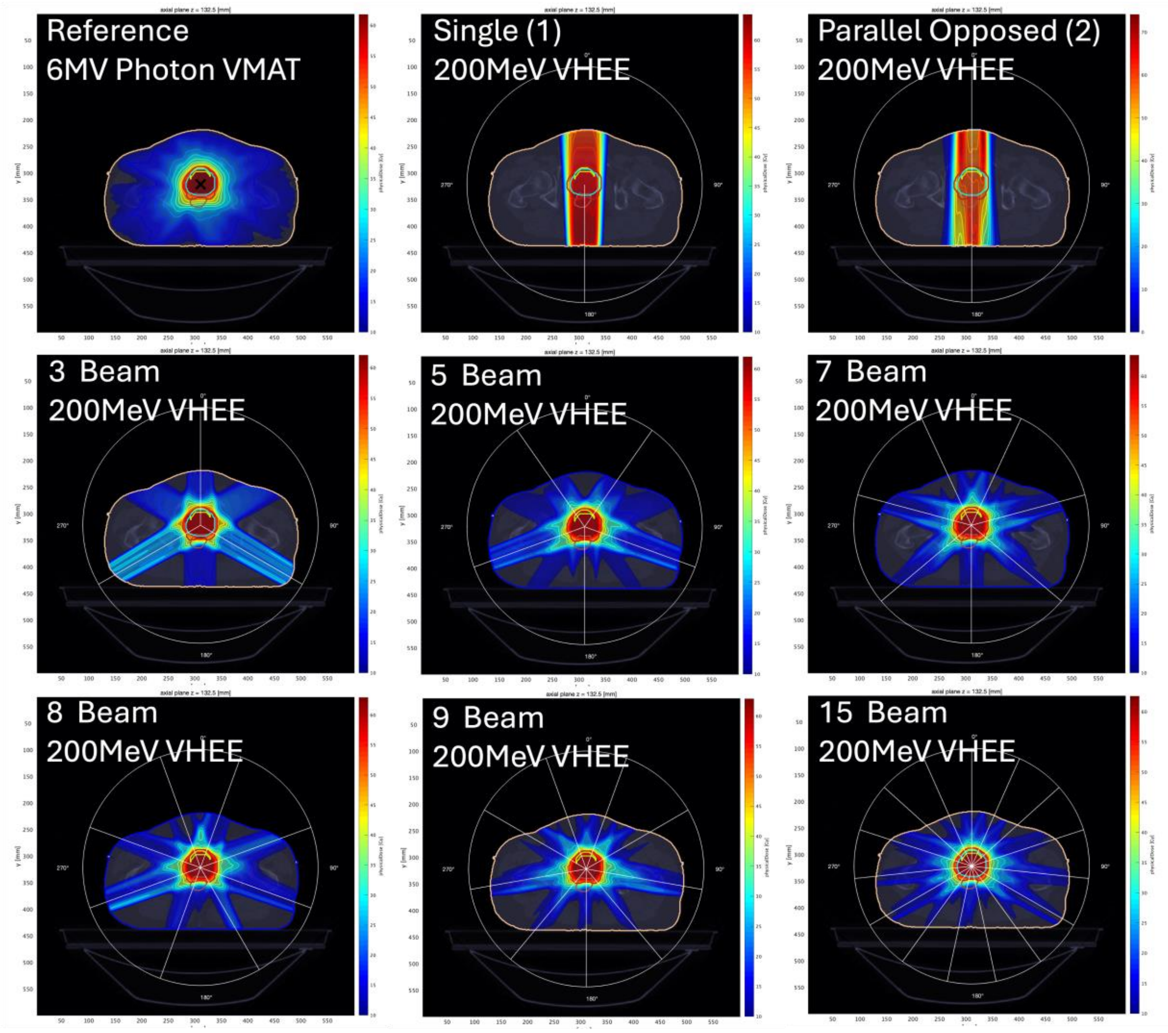

Supplementary Figure 3: Examples of a single-beam (1), parallel opposed beams (2), and configurations with 3, 5, 7, 9, and 15 beam arrangement for Prostate cohort.

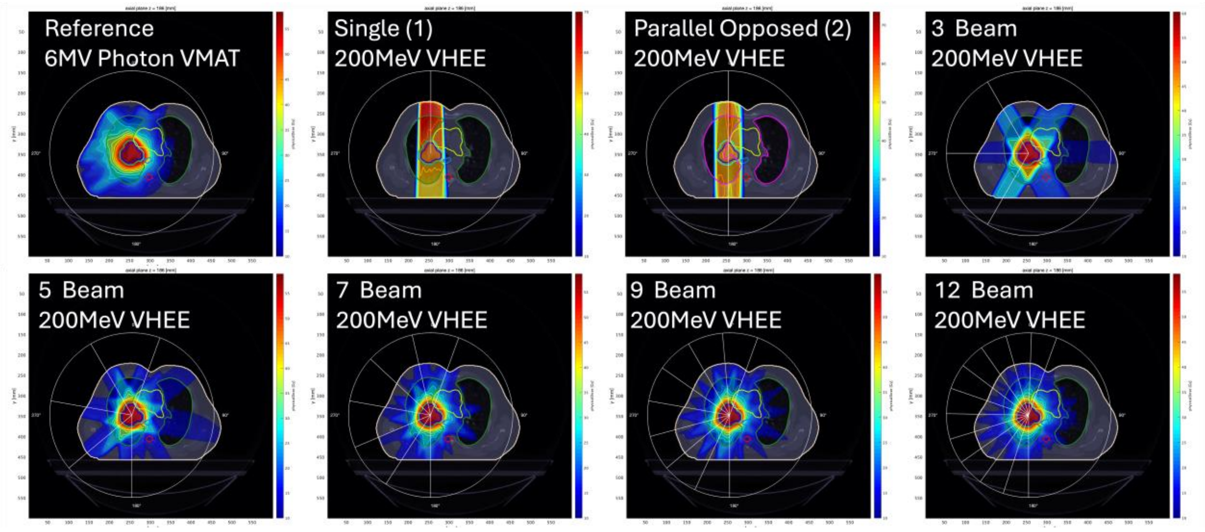

Supplementary Figure 4: Examples of a single-beam (1), parallel opposed beams (2), and configurations with 3, 5, 7, 9, and 12 beam arrangement for Lung cohort.

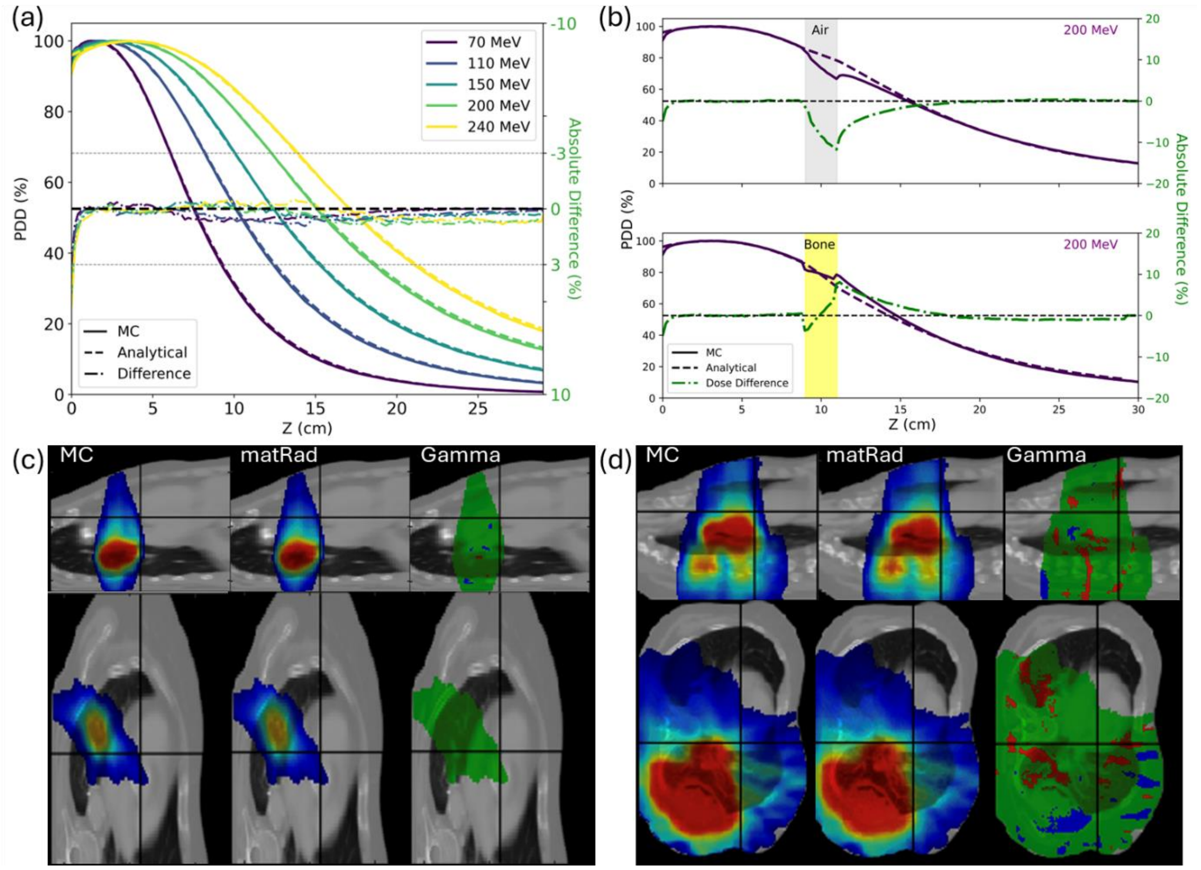

Supplementary Figure 5: Comparison of Analytical versus Monte Carlo (MC) PDD Curves for VHEE Beams at Various Energies with Depth (z). (a) displays PDD curves for 70, 110, 150, 200, and 240 MeV beams at varying depths, presenting MC results with solid lines and analytical model results with dashed lines, including absolute differences. (b) offers a detailed comparison for a 200 MeV VHEE beam featuring a 2 cm wide air (upper) and bone (lower) insert; density changes are highlighted in grey and yellow, respectively. In (c) and (d), dose distributions for two lung cancer patients treated with VHEE beams are compared between Monte Carlo (MC), matRad, and Gamma analysis. The top row of both (c) and (d) shows coronal views, while the bottom row of (c) presents sagittal views, and the bottom row of (d) shows axial views. Global gamma pass rates with 2%/2mm criteria are 96.7% for the first patient plan in (c) and 92.7% for the second patient plan in (d).

Spot spacing (σ)	PTV $D_{95\%}$ (Gy) Mean (range)	%	PTV D_{1cm^3} (Gy) Mean (range)	%	HI Mean (range)	Mean n Spots per 100cm^3	Mean time (min)
1.0	53.25 (53.04 - 53.41)	-	55.28 (55.15 - 55.49)	-	0.04 (0.03 - 0.04)	422	84.72
1.5	53.25 (52.98 - 53.39)	-0.2%	55.35 (55.19 - 55.54)	0.2%	0.04 (0.03 - 0.04)	208	38.90
2.0	53.07 (52.56 - 53.34)	-0.4%	56.09 (55.17 - 57.72)	1.4%	0.05 (0.03 - 0.08)	117	28.35
3.0	50.59 (48.80 - 51.71)	-5.1%	59.96 (57.99 - 62.86)	8.5%	0.16 (0.11 - 0.24)	52	12.62

Supplementary Table 1 : Summarises PTV metrics for spot spacings (σ) of 1.0, 1.5, 2.0, and 3.0. Metrics include D95% (Gy), D1cm3 (Gy), Homogeneity index (HI), mean spots per 100 cm3, and mean time (min).

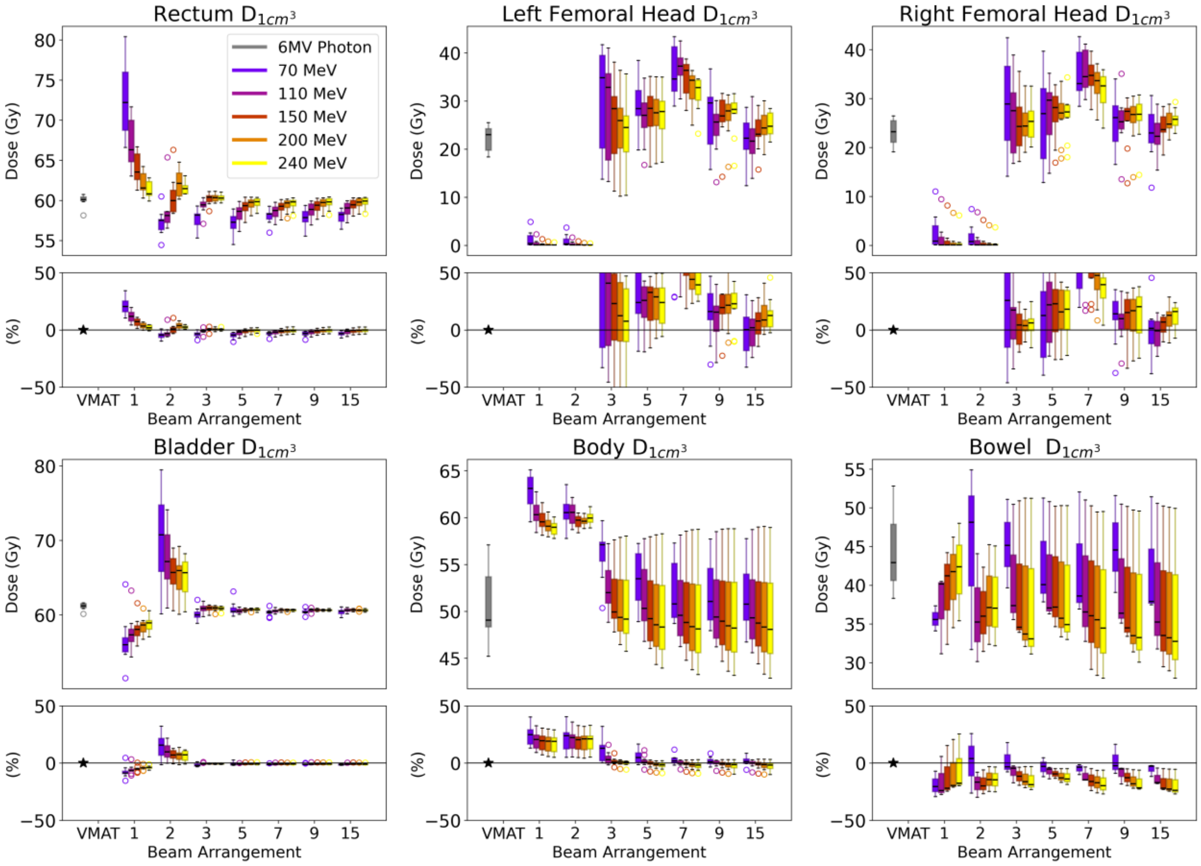

Supplementary Figure 6: The figure displays box-and-whisker plots comparing 400 dose distributions between 6 MV Photon VMAT and VHEE across various beam numbers (Single, Parallel-opposed, 3, 5, 7, 9, 12) and energies (70, 110, 150, 200, and 240 MeV). The VHEE plans were generated with a spot size of σ =4 mm and a spot spacing of 1.5σ. It presents dosimetric outcomes (D_{1cm^3}) for critical OARs: rectum, bladder, body, R/L Femoral heads and Bowel. Additionally, difference plots are included to visualise dosimetric deviations for OARs between the two modalities, highlighting relative and absolute differences across configurations.

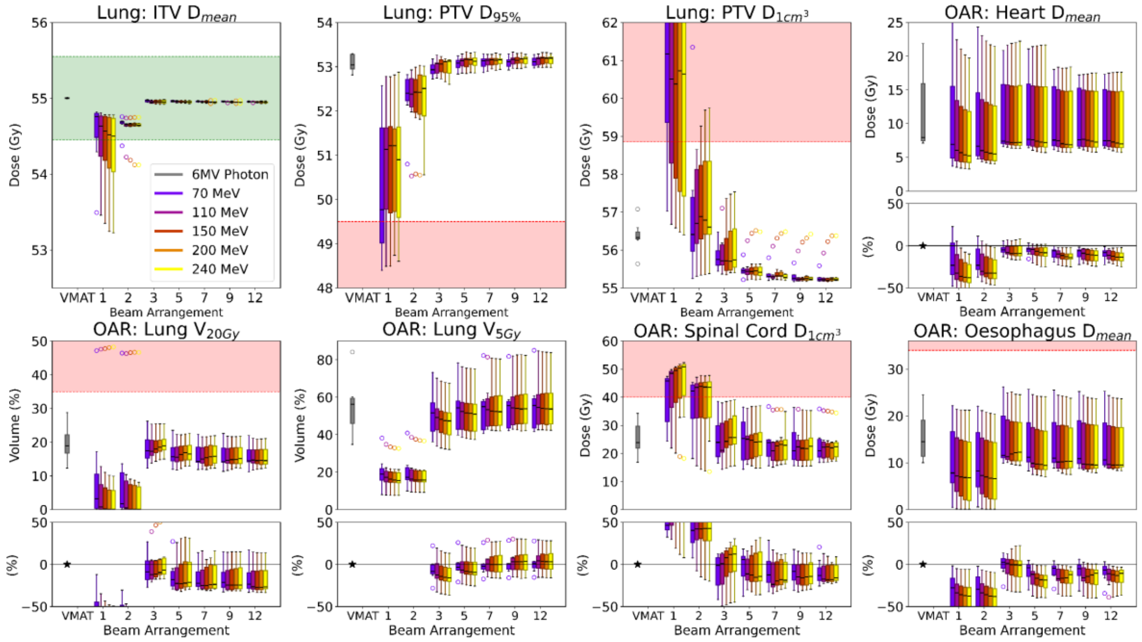

Supplementary Figure 7: Displays box-and-whisker plots comparing dose distributions for mid-line PTVs between 6 MV Photon VMAT and VHEE across different beam numbers (single, parallel-opposed, 3, 5, 7, 9, 12) and energies (70, 110, 150, 200, and 240 MeV). It presents target volume statistics (ITV Dmean, PTV D95%, and PTV D1cc), along with dosimetric outcomes for critical OARs, including Lung V20Gy & V5Gy, Heart Dmean, Oesophagus Dmean, and Spinal Cord D1cm³, expressed as relative shifts from the photon VMAT plan.

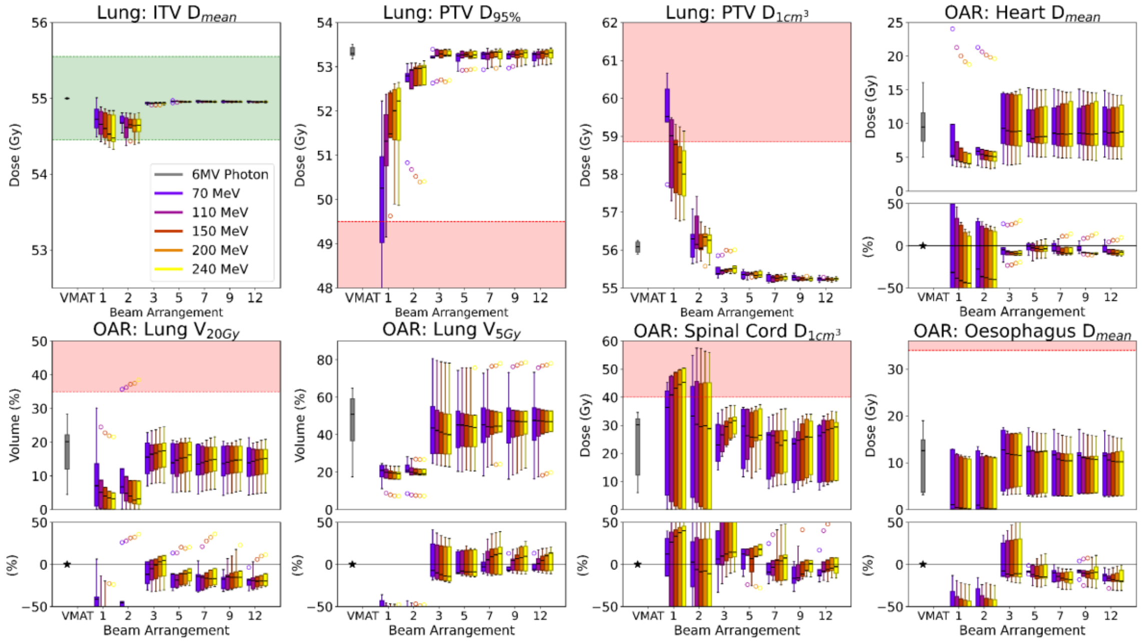

Supplementary Figure 8: Displays box-and-whisker plots comparing dose distributions for superficial PTVs between 6 MV Photon VMAT and VHEE across different beam numbers (single, parallel-opposed, 3, 5, 7, 9, 12) and energies (70, 110, 150, 200, and 240 MeV). It presents target volume statistics (ITV Dmean, PTV D95%, and PTV D1cc), along with dosimetric outcomes for critical OARs, including Lung V20Gy & V5Gy, Heart Dmean, Oesophagus Dmean, and Spinal Cord D1cm³, expressed as relative shifts from the photon VMAT plan.
